# Acellular carotid scaffold and evaluation the biological and biomechanical properties for tissue engineering

**DOI:** 10.34172/jcvtr.32899

**Published:** 2024-03-13

**Authors:** Farina Rashidi, Mehdi Mohammadzadeh, Arash Abdolmaleki, Asadollah Asadi, Mehrdad Sheikhlou

**Affiliations:** ^1^Department of Biology, Faculty of Science, University of Urmia, Urmia, Iran; ^2^Department of Biophysics, Faculty of Advanced Technologies, University of Mohaghegh Ardabili, Namin, Iran; ^3^Department of Biology, Faculty of Science, University of Mohaghegh Ardabili, Ardabil, Iran; ^4^Department of Engineering Sciences, Faculty of Advanced Technologies, University of Mohaghegh Ardabili, Namin, Iran

**Keywords:** Acellular artery, Chemical decellularization, Carotid, Regenerative medicine, Scaffold

## Abstract

**Introduction:**

The issues associated with the limitation of appropriate autologous vessels for vascular reconstruction via bypass surgery highlight the need for new alternative strategies based on tissue engineering. The present study aimed to prepare decellularized scaffolds from ovine carotid using chemical decellularization method.

**Methods:**

Ovine carotid were decellularized with Triton X-100 and tri-n-butyl phosphate (TnBP) at 37 °C. Histological analysis, biochemical tests, biomechanical assay and biocompatibility assay were used to investigate the efficacy of decellularization.

**Results:**

Decellularization method could successfully decellularize ovine carotid without leaving any cell remnants. Scaffolds showed minimal destruction of the three-dimensional structure and extracellular matrix, as well as adequate mechanical resistance and biocompatibility for cell growth and proliferation.

**Conclusion:**

Prepared acellular scaffold exhibited the necessary characteristics for clinical applications.

## Introduction

 Coronary artery disease (CAD) and peripheral vascular disorders are prominent causes of morbidity and mortality worldwide, with significant socioeconomic consequences.^1^ For example, CAD is the world’s third leading cause of death, accounting for 17.8 million deaths annually.^2,3^ Angiography is the most accurate diagnostic method for vascular disorders, and depending on the severity of the disease, medical therapy, angioplasty, stenting, or bypass grafting may be required.^4-6^ Bypass grafting is the most effective treatment for repairing damaged blood vessels and restoring blood flow.^7^ In this method, the surgeon creates a bypass by harvesting a healthy autologous artery or vein from another part of the body, primarily the saphenous vein, and redirecting blood around the blocked portion of the blood vessel.^7-9^ However, appropriate vessels for autograft are not always accessible due to previous vascular grafting, vasculopathy, or graft size mismatch.^10^ Furthermore, autologous vascular harvesting can cause complications at the donor site. As a result, artificial blood vessel grafts such as expanded polytetrafluoroethylene (ePTFE) tubes have been developed as alternatives to autologous blood vessel grafts.^11,12^ Nevertheless, there are some disadvantages to using artificial vessels, such as thrombosis, calcification, intimal hyperplasia, inflammation, and immune reaction.^13,14^ In this regard, acellular scaffolds derived from the decellularization of allogeneic or xenogeneic vessels appear to be appropriate substitutes for vascular autografts and artificial vascular conduits.^9,15^ During vascular decellularization, antigenic cellular components are removed, while extracellular matrix components such as collagen, elastin, and glycosaminoglycan (GAG) are mostly preserved.^16^ As a result, decellularization eliminates the immune response^17,18^ while preserving the vessel’s three-dimensional structure and mechanical properties.^19,20^ Furthermore, acellular scaffolds possess appropriate porosity, biochemical cues, and cell signaling components that promote host cell adhesion, proliferation, and differentiation, which are required for vascular wall remodeling and neovascularization.^21,22^ As a result of research on the preparation of decellularized xenograft vessels in experimental animal models,^23-26^ some decellularized xenografts, such as Artegraft® (bovine carotid artery), Solcograft® (bovine carotid artery), and ProCol® (bovine mesenteric vein), are now available for use in clinics.^27,28^ Despite these advancements, decellularized xenografts are still susceptible to thrombosis, intimal hyperplasia, and aneurysm, implying that more efforts to improve decellularized xenograft quality are necessary. As a result, our study aimed to prepare decellularized scaffolds from ovine carotid arteries using a suitable concentration of Triton X-100 and tri-n-butyl phosphate (TnBP). Furthermore, we also evaluated the morphology, mechanical features, and biocompatibility of decellularized vessels.

## Materials and Methods

###  Vascular harvesting

 Fresh ovine carotid arteries were obtained from a local slaughterhouse and transported to the lab in sterile ice-cold phosphate-buffered saline (PBS) containing 100 U/ml penicillin and 100 g/l streptomycin (Thermo Fisher Scientific). The specimens were cleaned in the lab to remove the blood, fat, and connective tissue and then kept in sterile PBS at -20 °C for decellularization.

###  Decellularization

 Ovine arteries were decellularized in standard flasks with Triton X-100 (Merck, Germany) and TnBP (Merck, Germany) at 37 °C. In brief, arteries were immersed in 1% (v/v) Triton X-100 solution and gently agitated for 15 hours. After being washed with distilled water, the specimens were immersed in a 1% (v/v) TnBP solution and continuously shaken for 4 hours. The samples were then washed with distilled water and incubated with 1% Triton X-100 for another 8 hours. Finally, in order to inactivate the toxic effects of decellularization materials organic soluble material was extracted via recirculating sterile water through ion exchange system for three times. Then artery samples were washed in PBS for 24 hours before being stored in antibiotic-containing PBS.^29,30^

###  Histological analysis

 Histological techniques were employed to investigate the efficiency of cell elimination and three-dimensional structure retention in decellularized arteries. In brief, specimens (n = 5) were fixed in 4% paraformaldehyde, embedded in paraffin, and cut into 5 µm-thick sections. After being deparaffinized with xylene and rehydrated with decreasing ethanol, samples were stained with Hematoxylin and Eosin (H&E)^31^ and 4,6-diamidino-2-phenylindole (DAPI)^32^ to assess cell elimination under light (Carl ZEISS-Axioskope 40, Germany) and fluorescence microscopy (BZ-X800, Keyence, Japan), respectively. Additionally, the collagen fibers were stained using Masson’s trichrome.^33^

###  Scanning electron microscopy (SEM)

 SEM was used to assess the ultrastructure of the native and decellularized arteries. First, the specimens (n = 5) were fixed for 24 hours at room temperature in 2.5% glutaraldehyde (Merck, Germany) diluted in PBS (pH 7.4). The specimens were washed three times in PBS and then dehydrated in an ascending ethanol gradient (50, 70, 90, and 100%) (Merck, Germany). After being air-dried, the specimens were coated with gold using a sputter coater and observed by the SEM (Hitachi, Tokyo, Japan).^34,35^

###  DNA content

 The total DNA in fresh and decellularized arteries was measured using a DNeasy Blood & Tissue Kit (Qiagen, Venlo, The Netherlands) to confirm complete cellular removal. Following the instructions, 20 mg of dry weight samples were digested with proteinase K at 56 °C and centrifuged to remove the protein fraction after adding a protein precipitation solution. The supernatant was then centrifuged with isopropanol and ethanol added. Finally, the sedimented pellet was rehydrated, and total DNA was quantified by measuring absorbance at 260 nm with a nanodrop spectrophotometer (Thermo Fischer Scientific, USA). The results are expressed as ng of DNA per milligram (mg) of dried tissue.^36^

###  Extracellular matrix (ECM) characterization

 To determine the effects of decellularization on the biochemical properties of decellularized arteries, total protein concentration and sulfated glycosaminoglycans (GAGs) of the ECM were measured. The total protein concentration was measured using the QuickZyme Total Protein Assay kit (QuickZyme Biosciences) according to the manufacturer’s protocol. In brief, native and decellularized specimens were hydrolyzed in HCl at 90 °C for 24 hours. The free hydrolyzed amino acids reacted with genipin to produce a blue-colored product, whose color intensity was proportional to protein concentration. Finally, the absorbance of colored products was measured at 570 nm with a VersaMax Spectrophotometer (Molecular Devices, USA) and compared to the absorbance of the standard kit control.

 In addition, the content of sulfated GAGs in native and decellularized arteries was determined using the Blyscan Sulfated Glycosaminoglycan Assay (Biocolor) according to the manufacturer’s instructions. Specimens were digested before being incubated with 1,9-dimethyl-methylene blue for 30 minutes. Following centrifugation, the insoluble GAG-dye complex was dissolved by incubating with the dissociation reagent, and absorbance was measured using a VersaMax Spectrophotometer at 513 nm and 656 nm (Molecular Devices, USA). GAG content was determined by comparing absorbances to a standard curve. The final values were expressed in milligrams of GAGs per milligram of dried tissue.

###  Biomechanical test

 A uniaxial tensile test was performed using a Zwick Roell (SANTAM-STM20, Tehran, Iran) to evaluate the effect of decellularization on the mechanical properties of decellularized arteries. In brief, the native and decellularized arteries (n = 5) were sectioned into 10 mm long rings with approximately the same diameters, and the initial cross-section area for each specimen was estimated. For analysis of ultimate tensile strength, the specimens were attached to two U-shaped stainless-steel grips on the tensile tester device and stretched at a constant rate of 20 mm/min to complete tensile failure. To keep the specimens moist, PBS was sprayed. Finally, the samples’ stress and strain values were estimated, and the stress-strain curve was produced. Stress is defined as force per unit area, and strain is the deformation of a solid caused by stress, which is computed by dividing the actual elongation by the initial length value. Furthermore, Young’s modulus (E) was calculated as the stress-strain ratio in the linear area of the stress-strain curve, which measures the samples’ tensile stiffness and viscoelastic behavior. Furthermore, Young’s modulus (E), which measures the samples’ tensile stiffness and viscoelastic behavior, was calculated as the ratio between the stress and strain in the linear area of the stress-strain curve.

###  Biocompatibility assay

 Adipose-derived stem cells (ASCs) were seeded on the luminal surface of the scaffolds to assess biocompatibility, and cell viability and proliferation were measured using the 3-(4,5-dimethylthiazohl-2-yl)-2,5-diphenyltetrazolium bromide (MTT) assay at 24, 48, and 72 hours after cell seeding. In brief, ASCs were isolated from the inguinal fat of adult male Wistar rats and characterized as previously described ^37^. The ASCs were cultured at a density of 1 × 10^7^ in 75 cm^2^ flasks (SPL, South Korea) containing 15 ml of low glucose Dulbecco’s modified Eagle’s medium (DMEM, Gibco, UK) supplemented with 10% (v/v) fetal bovine serum (FBS, Gibco, UK) and 100 U/ml penicillin/100 μg/ml streptomycin (Gibco, UK) at 37°C and 5% CO_2_. In our experiments, we used cells from passage four.

 On the other hand, decellularized arteries were cut into pieces (n = 3) with a length of 10 mm and an inner diameter of 4 mm. Afterward, the samples were placed in a 12-well plate (SPL, South Korea) containing DMEM with 10% FBS, 100 U/ml penicillin/100 g/ml streptomycin, and 1% Amphotericin B (Photericin B, Cipla, India) for 24 hours at 37 ºC and 5% CO_2. _The 10 µL ASCs suspension with a density of 3 × 10^5^/ml was then seeded onto the luminal surface of each decellularized artery specimen under a stereomicroscope (Carl ZEISS-C Stemi, Germany) and allowed to attach for 4 hours. The cell-artery combinations were incubated for 24, 48, or 72 hours in DMEM containing 10% FBS, 100 U/ml penicillin/100 g/ml streptomycin, and 1% Amphotericin B.

 The specimens were transferred to a 96-well plate containing DMEM supplemented with 20 μl MTT reagent (5 mg/ml; Merck, Germany) and incubated for 4 hours at 37 °C in the dark. Then, the medium was aspirated, and the specimens were incubated with 200 μl dimethyl sulfoxide (DMSO; Sigma, Germany) for one hour to dissolve precipitated formazan crystals. Finally, the solution absorbance, which corresponds to the number of viable cells, was measured using a microplate reader (model 550; Bio-Rad Laboratories, Hercules, CA, USA) at 570 nm ^38^. It is worth noting that ASCs cultured in the 12-well plate and decellularized arteries without ASCs were utilized as positive and negative controls, respectively. Every experiment was carried out in triplicate.

###  Statistical analysis

 All statistical analyses were performed by SPSS ver. 26 (SPSS Inc., Chicago, Illinois, USA) and results were presented as mean ± SD. One-way ANOVA was used for multiple comparisons between the groups, and Tukey-Post hoc analysis was performed to define the significant difference between them. A P-value of less than 0.05 was considered statistically significant.

## Results

###  Histology

**Figure 1:**
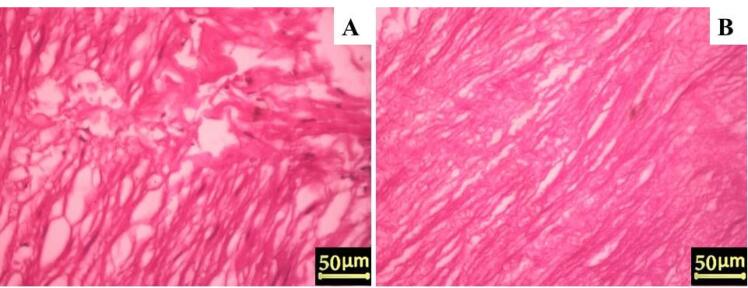
Hematoxylin-eosin staining of native and decellularized ovine carotid arteries. (A) Longitudinal section of native artery. The cell nucleus were stained in deep blue-purple and the pink color evidenced the cell cytoplasm (B) Longitudinal section of the decellularized artery that indicated cell nucleus elimination from decellularized specimens. Scale bar: 50 μm

**Figure 2:**
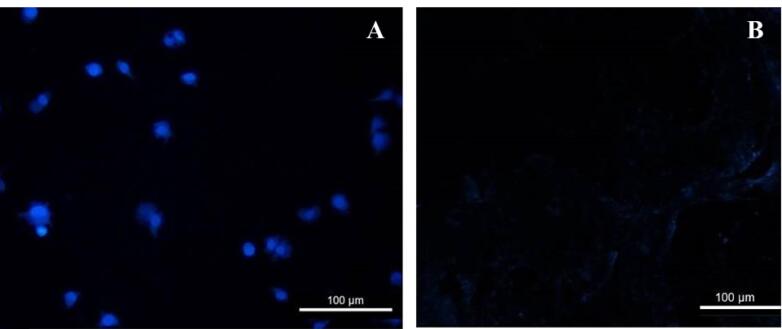
DAPI stained longitudinal-section of ovine carotid arteries were for illustrating the nuclei of the cells. (A) Native artery. The cell nucleus were stained as blue dots, (B) Decellularized artery. The cell nucleus were not detectable after decellularization. Scale bar: 100 μm

**Figure 3:**
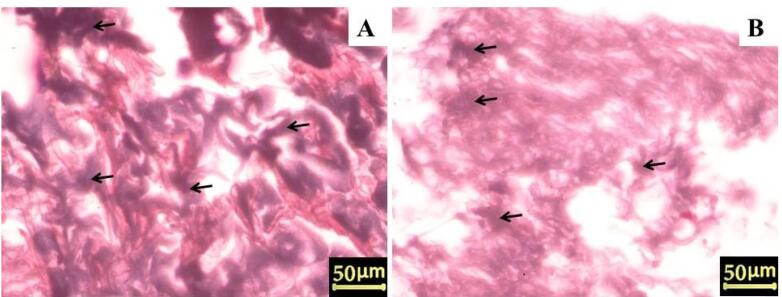
Masson’s trichrome staining of longitudinal-section of native and decellularized ovine carotid arteries for collagen fibers. (A) Native artery. The collagen is seen as dense wavy fibers (blue). (B) Decellularized artery. The collagen fibers were stained in blue (arrowhead). Scale bar: 50 μm

 The removal of cellular components and nuclei was confirmed using H&E and DAPI staining. H&E staining of native arteries revealed cell nuclei in deep blue-purple and cytoplasm in pink (Figure 1a). In contrast to native vessels, no residual cell nuclei or cytoplasm were found in decellularized vessels (Figure 1b). Furthermore, DAPI staining confirmed that the cell nuclei disappeared in the decellularized arteries (Figure 2a). DAPI staining marks the nuclei of cells in native arteries in blue dots (Figure 2b). On the other hand, the ECM of decellularized vessels was evaluated by staining collagen fibers with Masson’s trichrome. Collagens are visible in native arteries as dense blue wavy fibers, as shown in Figure 3a. The results showed that collagen fibers were well preserved in the decellularized vessels, despite appearing slightly less compact and more extended when compared to native arteries (Figure 3b). These findings suggest that the artery decellularization process was effective in removing the cell component and ECM retention.

###  SEM

**Figure 4:**
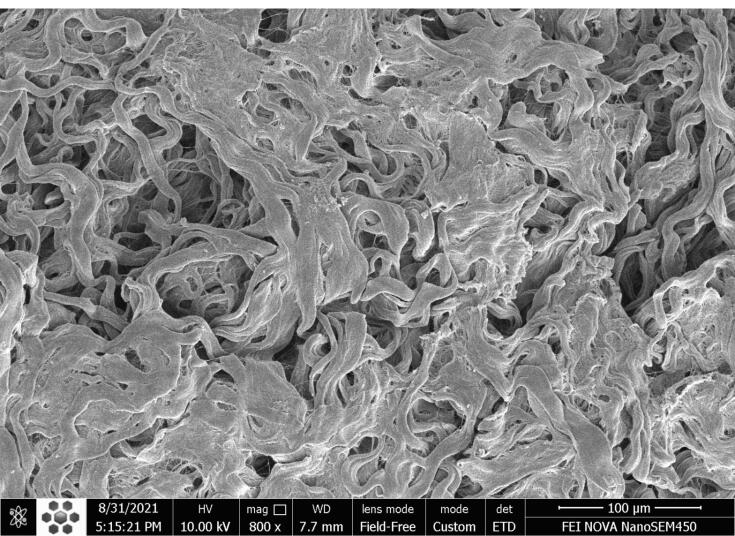
Scanning electron microscopy of longitudinal-section of decellularized arteries. Decellularized artery ultrastructure. (magnification, × 800; Scale bars 100 µm)

 The ultrastructure of the decellularized arteries was studied using SEM micrographs. The result showed that an intact layer of endothelial cells covered the luminal surface of native arteries, and the intimal, medial, and adventitial layers were tightly integrated (Figure 4). However, no cells were found on the luminal or adventitial surfaces of the scaffolds after decellularization. Furthermore, the intimal, medial, and adventitial layers of decellularized arteries were partially preserved, and the vascular wall became porous (Figure 4).

###  DNA, total protein, and sulfated GAGs content

**Figure 5:**
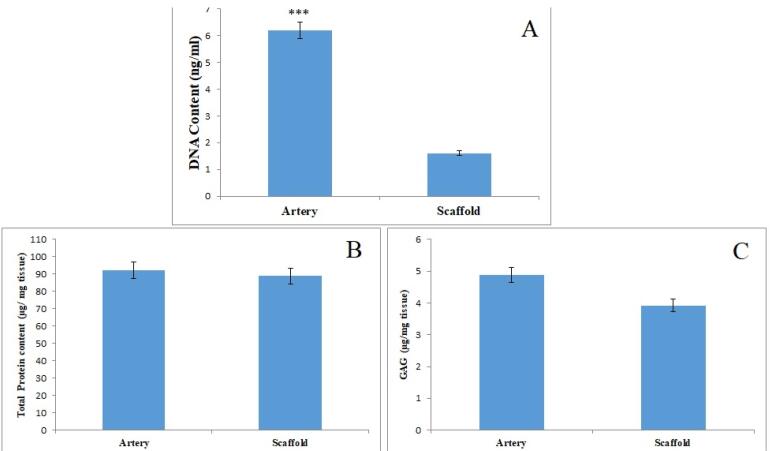
Confirming preservation of the ECM content after decellularization. Quantification of DNA content before and after decellularization Mean ± standard deviation (SD), n = 3, *** indicates *P* < 0.001 (A). Total protein and sulfated GAGs content evaluation showed no significant difference between decellularized and native arteries (B and C)

 DNA quantification was performed to determine the efficacy of the decellularization process. The amount of DNA extracted from the native and processed arteries was measured in this regard. The results revealed that DNA content was significantly reduced in decellularized arteries (^***^P < 0.001; Figure 5A) when compared to native vessels. This finding was consistent with the results of H&E and DAPI staining. Furthermore, total protein and sulfated GAGs content were quantified to assess the effects of decellularization on the ECM. These findings imply that there is no significant difference in total protein and sulfated GAGs content between decellularized and native arteries. As a result, the ECM of the arteries had not changed significantly during the decellularization process (ns, Figure 5B and C).

###  Biomechanical test

**Figure 6:**
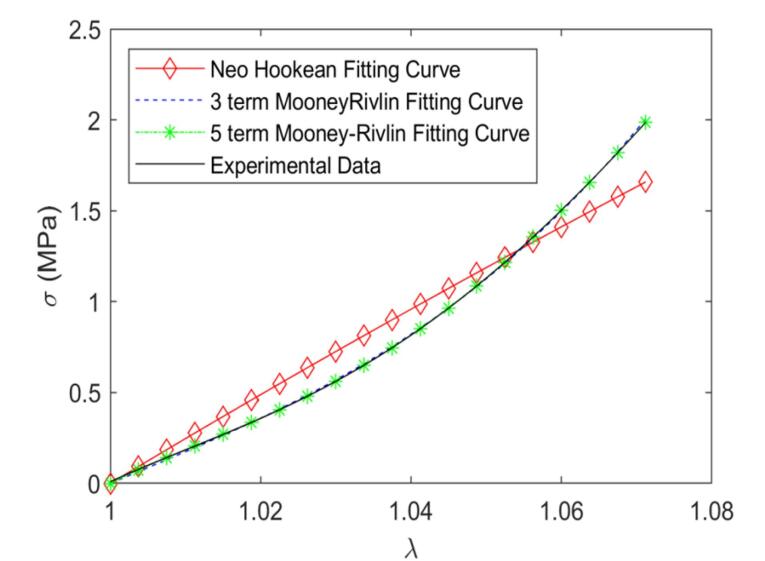
The stress-strain curve and fitted hyper elastic models for the native arteries

**Figure 7:**
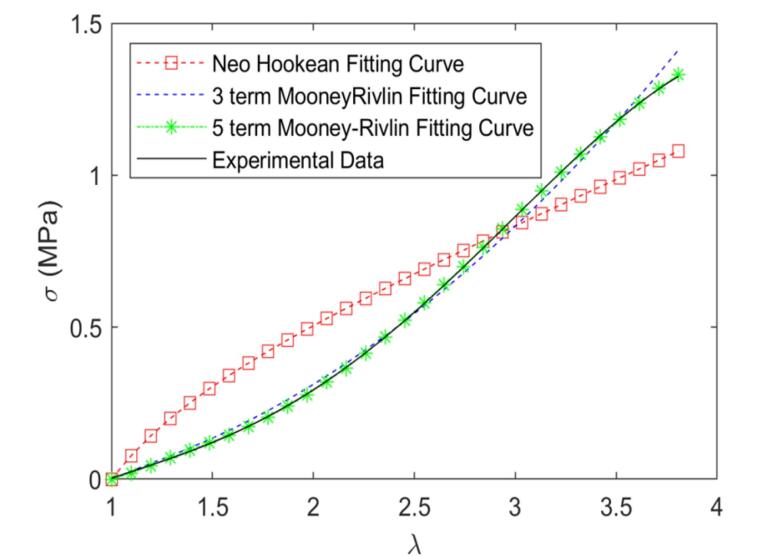
The stress-strain curve and fitted hyperelastic models for the decellularized arteries

 The uniaxial tension test was employed to quantify the nonlinear mechanical behavior and hyperelastic properties of the acellular arteries. Based on the outcomes of the samples’ uniaxial tensile tests, the coefficients of the strain energy density function of the proposed hyperelastic models were estimated using the non-linear curve fitting method. These coefficients can be employed to predict the mechanical response of decellularized arteries under various loading conditions. Table 1 shows the coefficient values of hyperelastic models for native and decellularized arteries. Figures 6 and 7 depict the stress-strain curves and fitted hyperelastic models for the native and decellularized arteries, respectively. As seen in the diagrams, the Neo Hookean model was not capable of predicting the nonlinear behavior of the native and decellularized arteries under uniaxial tensile conditions. As shown in Figure 6, because the vessel deformation range is limited, both the three-term and five-term Mooney-Rivlin strain energy density functions predict the behavior of native arteries in the range of our test. Figure 7 shows that, while the three and five-term Mooney-Rivlin strain energy density functions have comparable efficiency for small deformations of decellularized arteries, the five-term function performs better in our test range for large deformations. As a result of the deformation and more extensive nonlinear behavior of the decellularized artery compared to the native vessels, the five-term Mooney-Rivlin model, which includes higher-order nonlinear terms, can better predict the decellularized arteries’ nonlinear hyperelastic behavior.

**Table 1:** Coefficients value of hyperelastic models for native and decellularized arteries.

**Hyperelastic models**	**Coefficients**	**Native arteries**	**Decellularized arteries**
Neo Hookean model	*C*_10_	4.156	0.1442
	*sse*	8.8201	24.9670
Three-term Mooney-Rivlin model	*C*_10_	-0.8304	0.0490
	*C*_01_	3.7545	2.2204 × 10^-14^
	*C*_11_	85.2734	0.0178
	*sse*	0.0247	0.5169
Five-term Mooney-Rivlin model	*C*_10_	-5.5600	0.2591
	*C*_01_	8.5371	-0.2388
	*C*_11_	100.00	0.1359
	*C*_20_	-94.1680	-0.0230
	*C*_30_	100.00	-0.2164
	*sse*	0.0199	0.0048

###  Biocompatibility with cells

**Figure 8:**
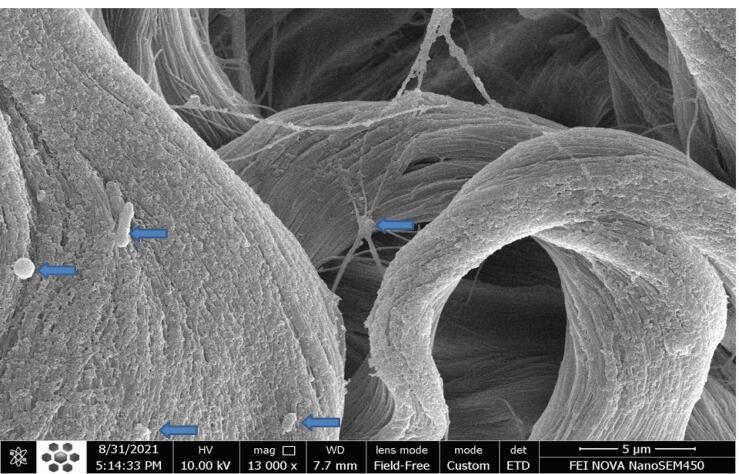
Scanning electron microscopy 7 days after cell seeding on decellularized arteries. (magnification, × 13000; Scale bars 5 µm)

**Figure 9:**
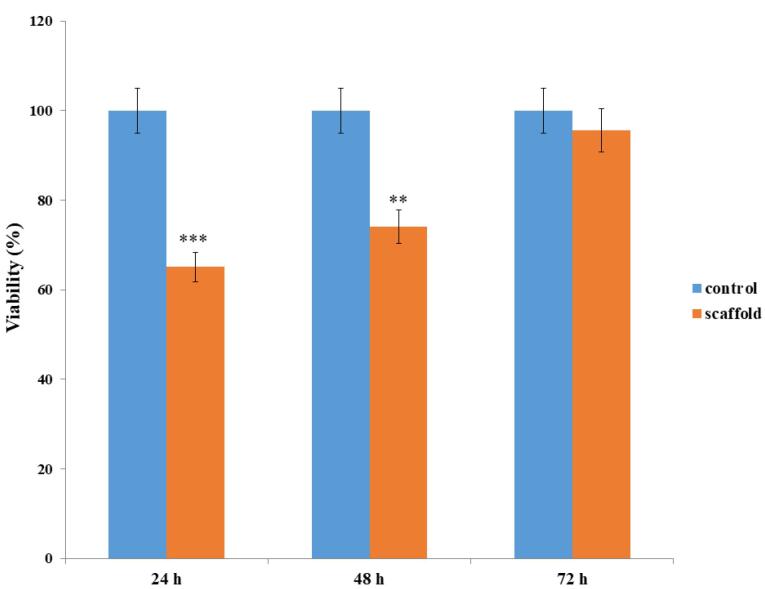
MTT assay for evaluation of ASCs viability on the luminal and adventitial surface of decellularized arteries. Results showed that the viability and proliferation of the ASCs seeded on decellularized arteries significantly increased by increasing culture time compared to the control group

 The cell retention capacity of decellularized arteries was estimated using SEM micrographs and the MTT assay. SEM micrographs revealed that ASCs adhered to and expanded on the luminal surfaces of decellularized arteries 7 days after cell seeding (Figure 8). Furthermore, the MTT assay, which measures the metabolic activity of cells, revealed that the viability and proliferation of ASCs seeded on decellularized arteries were significantly lower than ASCs cultured in 96-well plates (controls) at 24 and 48 hours (*P* < 0.05; Figure 9). However, 72 hours after cell seeding, there was no significant difference between the ASCs-scaffold group and the control group, demonstrating proper cell adhesion and proliferation in the decellularized arteries. The MTT assay results confirmed the SEM findings.

## Discussion

 In the case of CAD and peripheral vascular disorders, bypass grafting with autologous blood vessels is still the best option for restoring blood flow.^7^ However, clinical application of autologous vessels is limited due to a lack of suitable donor veins or arteries, donor site morbidity, and a size mismatch between the donor and recipient vessels.^10,39^ Due to these limitations, efforts were made to develop alternatives to autologous vessels for vascular bypass grafting. These alternatives should have a confluent endothelium and smooth muscle cells, as well as enough mechanical integrity and elasticity to tolerate systemic arterial pressure.^27,40^ In this regard, decellularized vessels derived from human cadavers or animals are promising candidates for regenerative medicine.^41,42^ Furthermore, customizing these scaffolds with stem cells can improve their capacity for regeneration.^43,44^ Decellularized artery allografts or xenografts are promising alternatives to autologous blood vessels because decellularization removes cells while preserving the three-dimensional structure and ECM components of native arteries.^45,46^ Due to the retention of native vessel ECM, the acellular vessels can promote cell attachment, proliferation, and differentiation during regeneration.^40,41^ Acellular vessels also contain molecular cues, such as growth factors, that promote host cell migration and neovascularization.^27^ Furthermore, decellularization prevents immunological rejection of acellular scaffolds by removing antigenic cellular components.^13^ Despite these advantages, thrombosis, aneurysms, and intimal hyperplasia are among the problems in decellularized vessels.^30^

 Decellularization methods vary in efficiency, and there is no standard protocol in this field.^16^ As a result, it is preferable to seek the best decellularization method that, while removing all cellular components, causes the minimum change in the three-dimensional structure and ECM of the vascular tissue.^47,48^ Furthermore, each decellularization method has its disadvantages. For example, physical methods such as sonication, freeze-thaw cycles, and agitation can degrade ECM,^49,50^ while enzymatic techniques can remove important ECM components such as laminin, fibronectin, and elastin. Trypsin is one of the most frequently utilized decellularization enzymes, along with nucleases, lipases, collagenases, dispase, α -galactosidase, and thermolysin.^47,51^ Among all the decellularization methods, chemical decellularization protocols using detergents are one of the simplest, inexpensive, and most widely used methods. Detergents could disrupt the cell membrane and cellular compartments and also separate DNA from proteins.^20^ The most commonly used detergents are Triton X-100 as a non-ionic detergent and sodium dodecyl sulfate (SDS) as an ionic detergent.^52^ Triton X-100 is a mild detergent that breaks down lipid-lipid and lipid-protein interactions but not protein-protein interactions.^53^ On the other hand, although SDS can eliminate cellular components by disbanding the cytoplasm and nucleus, it can also damage the ECM.^54^ As a result, we aimed to decellularize ovine carotid arteries with a combination of triton X-100 and TnBP, as well as investigate their chemical and mechanical properties, and also their biocompatibility with cells. TnBP is an amphiphilic solvent and extractant with a polar head and nonpolar tails.^55^ TnBP, on the other hand, is weaker than SDS and reduces the risk of ECM damage.

 According to our results, H&E and DAPI staining confirmed the elimination of cell nucleus and cellular components from decellularized arteries. Furthermore, SEM analysis revealed that endothelial cells had been removed from the luminal surface of decellularized arteries. The DNA quantification assay also provided further evidence of removing the DNA content from the decellularized arteries. According to the results, the DNA content of decellularized arteries was reduced to less than 50 ng/mg of dried tissue, which was within the standard range for decellularized tissues.^47^ Masson’s trichrome staining and SEM micrographs revealed that the three-dimensional structure and collagen fibers of decellularized arteries were properly preserved when compared to native vessels. Many studies show the importance of collagen as the main ECM protein in promoting cell proliferation and differentiation. In addition, total protein and sulfated GAG measurements revealed no significant change in the chemical composition of the ECM. On the other hand, the integrity of the ECM typically determines the mechanical characteristics of decellularized tissues.^56^ The biomechanical test results also indicated that the decellularized scaffolds were stable during the uniaxial tensile test. The mechanical resistance of scaffolds is crucial because the appropriate mechanical properties of the acellular arteries are necessary for physical integrity and elasticity against systemic arterial pressure to avoid aneurysm formation. Furthermore, acellular arteries must be capable of withstanding suture strength after bypass surgery. Other microscopic analyses and the MTT assay revealed that the luminal surface of the decellularized arteries could provide an appropriate substrate for the adherence, growth, and proliferation of the seeded ASCs. Evidence suggests that the basal lamina layer of the luminal surface can control cell attachment, proliferation, and migration by interacting with seeded cells via glycoproteins and proteoglycans.^57^ It is conceivable that paracrine pathways might boost the likelihood of differentiation if cells were positioned correctly in the scaffolds during regeneration.^58^ Furthermore, previous in vitro and in vivo research has shown that mesenchymal stem cells (MSC) can differentiate into vascular cells such as endothelial cells and smooth muscle cells.^59,60^

 Despite promising results, our current study also has some limitations. Firstly, we did not assess the effects of different Triton and TnBP concentrations on vascular decellularization. Additionally, the differentiation process of the cells implanted in the acellular arteries was not investigated. Also, if immunohistochemical studies had been conducted, we could have provided a more accurate estimate of the ECM composition.

## Conclusion

 In summary, our results demonstrated that the combination of 0.1% Triton X-100 and 0.1% TnBP could successfully decellularize ovine carotid arteries without leaving any cell remnants. The decellularized scaffolds showed minimal destruction of the three-dimensional structure and extracellular matrix, as well as adequate mechanical resistance and biocompatibility for cell growth and proliferation. The advancement of regenerative medicine allows for the clinical application of this scaffold for CAD and peripheral vascular disorders. In future works cross-sectional microscopic image should be prepared for showing vessels structure.

## Acknowledgements

 The authors thank the University of Urmia for funding this project.

## Competing Interests

 The authors declare that there is no conflict of interest.

## Ethical Approval

 All experimental procedures were conducted in accordance with the European Union Council Directive of November 24, 1986(86/609/EEC), and followed the guidelines developed by the ethical committee at the University of Urmia with ethic number: IR-UU-AEC-33304.

## Funding

 This study was supported by University of Urmia.
